# Automatic Key Update Mechanism for Lightweight M2M Communication and Enhancement of IoT Security: A Case Study of CoAP Using Libcoap Library †

**DOI:** 10.3390/s22010340

**Published:** 2022-01-03

**Authors:** Wen-Chung Tsai, Tzu-Hsuan Tsai, Te-Jen Wang, Mao-Lun Chiang

**Affiliations:** 1 Department of Information and Communication Engineering, Chaoyang University of Technology, Taichung City 413310, Taiwan; s10930608@gm.cyut.edu.tw; 2 Smart System Institute, Institute for Information Industry, Taipei City 10622, Taiwan; roytwang@iii.org.tw; 3 Bachelor Degree Program of Artificial Intelligence, National Taichung University of Science and Technology, Taichung City 40401, Taiwan

**Keywords:** Internet of Things, information security, machine to machine, constrained application protocol

## Abstract

The ecosystem for an Internet of Things (IoT) generally comprises endpoint clients, network devices, and cloud servers. Thus, data transfers within the network present multiple security concerns. The recent boom in IoT applications has accelerated the need for a network infrastructure that provides timely and safe information exchange services. A shortcoming of many existing networks is the use of static key authentication. To enable the use of automatic key update mechanisms in IoT devices and enhance security in lightweight machine-to-machine (M2M) communications, we propose a key update mechanism, namely, double OTP (D-OTP), which combines both one-time password (OTP) and one-time pad to achieve an IoT ecosystem with theoretically unbreakable security. The proposed D-OTP was implemented into the Constrained Application Protocol (CoAP) through the commonly used libcoap library. The experimental results revealed that an additional 8.93% latency overhead was required to obtain an unbreakable guarantee of data transfers in 100 CoAP communication sessions.

## 1. Introduction

Business opportunities in the Internet of Things (c.f., IoT in Appendix A) industry are increasing rapidly, and according to a report by International Data Corporation (IDC) FutureScapes [1], among the 10 fields predicted to lead in the industry, security is ranked highest. However, IoT security-related concerns must be carefully discussed and addressed. Currently, the introduction of 5 G technology has accelerated the development of the IoT. However, developing a network infrastructure that can adequately guarantee security and meet the requirements of lightweight machine-to-machine (M2M) transmissions in wireless networks remains a challenge for researchers and developers [2]. Furthermore, an increase in potential applications of the IoT has necessitated acceleration in the development of a network infrastructure to meet the demands of massive machine-type communication for the tremendous number of IoT devices. Efforts toward achieving a secure IoT ecosystem are moving toward development of an infrastructure that can support massive connections and diverse applications without sacrificing information security.

**Figure 1:**
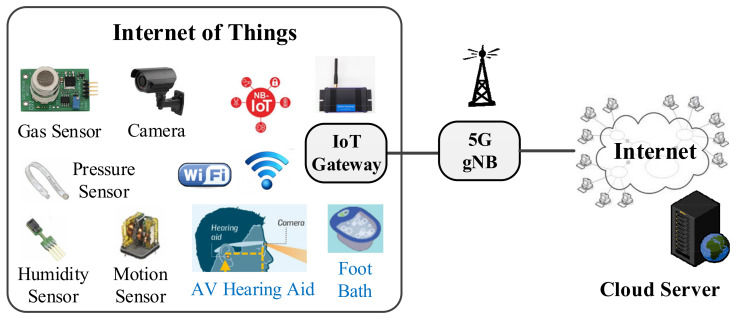
Potential applications of IoT within 5 G networks.

As Figure 1 shows, an IoT ecosystem generally comprises endpoint clients (e.g., sensing nodes), network devices (e.g., routers), and cloud servers (e.g., workstations); however, within this system, the data transfers that occur between clients and servers present multiple information security concerns. To address this, network security researchers have worked to increase the robustness of encryption and decryption algorithms. Furthermore, if the applied security mechanism can be improved to suit the characteristics of different applications, it could achieve more optimal performance metrics for cost, speed, and security. For example, Adeel et al. [3] recently provided a lightweight chaotic encryption scheme for transmitting information of audiovisual (AV) hearing aids in real time. Besides, this study introduces secure IoT applications of sensor bracelets, heated socks, and foot baths in Section 3.3. Therefore, establishing timely and safe information exchange services among IoT devices is crucial.

The Constrained Application Protocol (CoAP) [4] is intended for use in IoT devices. The CoAP was designed to meet specialized application requirements, such as low throughput and low power, in lightweight M2M communications and IoT devices. Therefore, we selected the CoAP as the protocol applied in our experiments. Several unique implementations of CoAP have been accomplished, such as with libcoap [5], a source code archive that can be cloned from GitHub. In this study, we modified the designs of the CoAP server and CoAP client provided in libcoap version 4.2.1. Moreover, we used OpenSSL version 3.0 [6] to enable encryption in our implementation.

However, working from within an existing network may require researchers to use a static key authentication structure, such as the pre-shared key (PSK) used in OpenSSL [6]. By contrast, a one-time password (OTP) [7] is a system in which a password is valid for only one login session on a computer or for a single data transfer over a network. Therefore, to avoid the shortcomings associated with static key authentication, we applied OTP with CoAP in our previous work [8]. Another technique, one-time pad [9], refers to a key that can only be used once (i.e., nonrepeating) for encrypted operations. One-time pad is an encryption operation technique in classical cryptography and has been reported by Shannon [10] to be theoretically unbreakable. Recent implementations of OTP, such as those of [11,12,13] served as references for the implementation of our own proposed key update mechanism in which we aimed to simultaneously execute both OTP and one-time pad in IoT devices as a pair, namely, D-OTP (Double OTP).

This paper is organized as follows: in Section 2, the background of security-related key systems is explained. In Section 3, the design methodology, including the proposed key update mechanism of the applied IoT ecosystem, is introduced. In Section 4, the experimental results with performance analyses are presented and described. In Section 5, we discuss the results of our experiments, and a conclusion is drawn in Section 6.

## 2. Background

In this section, we review and evaluate existing network security issues, especially those of key management methods. We next provide a basic explanation of cipher algorithms. Finally, we discuss the application layer protocol and nonrepeating rule of the keys used in this study.

### 2.1. PSK

PSKs are designed for communication between devices and, therefore, do not require an authentication server. For example, in a wireless network, such as Wi-Fi [14], each device encrypts the traffic datagram by deriving its encryption key from a PSK. However, the same PSK is repeatedly used for different data transfers, which can leave PSKs vulnerable to password cracking if users have a weak password or passphrase. For example, as reported in one study [15], the name and length of the service set identifier (SSID) are used to seed Wi-Fi Protected Access (WPA) password hashes. However, rainbow tables have been created for the top 1000 SSIDs and common passwords; such a table can thus be employed to rapildy crack a WPA-PSK. Generally, PSKs should be replaced regularly, especially if a device has been lost, or when a user’s right to use a network has recently been restored. For IoT applications, an automatic key update mechanism is essential for enhancing the security of lightweight M2M communications.

### 2.2. Key Exchange

To change keys for communication between devices, the Diffie–Hellman (DH) algorithm [16] is a method of securely sharing a common cryptographic key for symmetric encryption over a public channel. Relevantly, another asymmetric cryptographic system that uses pairs of keys consisting of a public key and a private key, such as Rivest–Shamir–Adleman (RSA) [17] in which each device requires keeping the private key and the public key, can be distributed without compromising security [18]. Another key exchange method is quantum key distribution (QKD), which allows two parties (commonly called Alice and Bob) to share a common key for encryption via a quantum channel (i.e., QKD link) [19]. The first QKD protocol, which was known as BB84 [20]. By which, if an eavesdropper (conventionally called Eve) tries to steal the key, the communicators (i.e., Alice and Bob) will detect her using appropriate quantum laws [21]. Accordingly, the PSKs can be updated frequently between devices by taking advantage of the mentioned key exchange methods. As a result, transmission delays are required for the key exchanges, especially for the QKD based system [22].

In comparison with our proposed D-OTP mechanism, the PSKs are updated in each device without exchanging any information between each other. In brief, the keys are generated by a pseudorandom number generator (PRNG) using a seed as the identical serial number (SN) recorded in the read-only memory (ROM) of the communication devices. Section 3.2 will provide a detailed introduction.

### 2.3. Ciphering Methods

In data encryption, a stream cipher is a type of algorithm that uses a symmetric key or a single key that is used to both encrypt and decrypt. Generally, a stream cipher uses a PRNG to generate a key data stream during encryption [23]. Through this, the key data stream is sequentially encrypted with a plaintext data stream to obtain a ciphertext data stream. For example, the Rivest Cipher 4 (RC4) sequentially performs an exclusive-OR (XOR) operation on each byte of the plaintext data stream and each byte of the key data stream; after the operation, one byte of ciphertext data can be obtained in sequence and then used to produce a ciphertext data stream. Because implementation of RC4 is relatively simple and direct, as was illustrated by [24,25], thus stream encryption is suitable for real-time transmission of audio and video streaming data within a network.

Block encryption is another type of symmetric key encryption algorithm; it is used, for example, in the Advanced Encryption Standard (AES), a popular specification for the encryption of datagrams established by the National Institute of Standards and Technology in 2001 [26,27]. The AES superseded the previous block cipher method, Data Encryption Standard (DES) [28], and is now commonly used worldwide in many network and communication standards. In terms of design, block cipher can also be used to encrypt data stream. However, block encryption requires a certain amount of data to be collected as a block before the encryption start. Thus, design and operation of block cipher are more complicated than that of stream cipher. In general, block encryption can provide better secure performance, it is more capable for encrypting data file transferred over the network.

### 2.4. TLS

TLS [29] is a cipher protocol designed to provide communications security over the transport layer of a computer network. TLS is commonly used in network applications. For example, it is typically used on websites to secure communication between servers and browsers. TLS version 1.2 supports AES–CCM (i.e., counter with CBC–MAC mode) and AES–GCM (i.e., Galois/counter mode) to provide privacy and data integrity between two communication devices, which may be a server and a client. Furthermore, when datagrams are secured using TLS, the connection becomes secure because of the symmetric cryptography that is employed to encrypt the data transfer. For this type of encryption, a public key is generated for each connection at the start of the session. The server and client negotiate the public key before the first byte of data is transmitted. However, the security mechanism for exchanging the public key between the server and the client is problematic. For M2M applications, especially those in a fixed network configuration, the public key can be preset in a communication pair of devices. Accordingly, we propose a design methodology especially suited for IoT devices.

### 2.5. CoAP and Secure IoT Transmission

**Figure 2:**
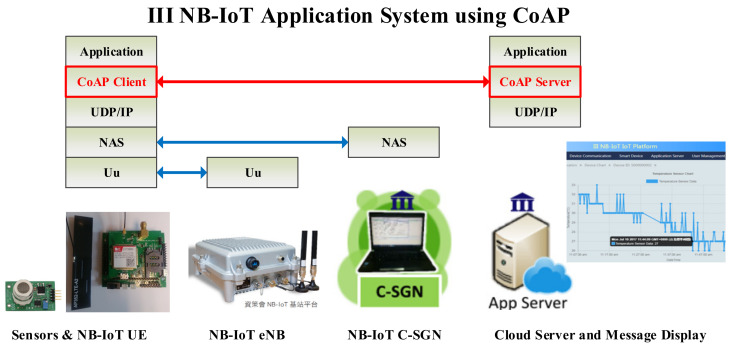
The III NB-IoT sensor system employs CoAP as the IoT communication protocol.

CoAP, defined in RFC 7252 [4], is intended for use in IoT devices, such as wireless sensor nodes, that may be resource-constrained with low power consumption and low computational capacity. CoAP can enable IoT devices to be accessed over the Internet through the transfer of datagrams using Transmission Control Protocol (TCP) or User Datagram Protocol (UDP) sockets. CoAP is designed to meet specialized application requirements, such as low throughput and low power. Low overhead and simple design are essential in lightweight M2M communications and IoT devices, both of which tend to be deeply embedded, have low memory, and have a simple microprocessor, owing to the Institute for Information Industry (III) that provided us with the narrowband Internet of things (NB-IoT)-based [30] sensor application system [31] as shown in Figure 2, in which CoAP is selected as the IoT communication protocol. The CoAP transmission service can use Datagram TLS (DTLS) [5] in either raw public key (RPK) mode or PSK mode for data encryption. However, both RPK and PSK are static. Therefore, we cooperated to develop the proposed automatic key update mechanism of D-OTP. For this reason, we selected to apply CoAP in this study. Section 3 “Design Methodology” will introduce the details.

### 2.6. CoAP Library of Libcoap

Some implementations based on the CoAP can be obtained from the Internet; libcoap [5] is easily available as a source code archive that can be cloned from GitHub. Moreover, libcoap is C implemented and supports TLS, including the GnuTLS, OpenSSL, and tinydtls frameworks. In this study, we modified the designs of the CoAP server and CoAP client provided in libcoap version 4.2.1. Furthermore, we used OpenSSL version 3.0 [6] to enable encryption in our implementation.

### 2.7. Nonrepeating PRNG

Our proposed D-OTP is a secure mechanism that provides theoretically unbreakable security, as reported by Shannon [10]. For this mechanism to be implemented, a nonrepeating PRNG (NR-PRNG), as used in [32], is required. A nonrepeating key is created by randomly selecting a nonrepeating element from a set of *n* elements. Most repeating keys, such as that of [33], have been developed on the basis of the Fisher–Yates shuffle algorithm [34]. However, this method places a heavy burden on computing performance for generating high entropy random numbers, and providing nonrepeating checks through the method requires a large amount of memory access [11]. Accordingly, we address this concern in our proposed design.

## 3. Design Methodology

We first introduce the proposed D-OTP key update mechanism and provide a general overview of different implementations of CoAP and PRNG. We then introduce the execution flow, including the CoAP server and CoAP client communication pair. Finally, we provide a description of the implemented key update mechanism and its operation flow.

### 3.1. OTP and One-Time Pad

An OTP is a password that is valid for only one login session on a computer or for a single data transfer over a network [7]. Through implementation of an OTP ecosystem, the static key authentication-related shortcomings of PSK can be avoided. OTP is commonly used for credit card transactions. When a credit card payment is completed, an OTP is randomly generated and sent to a user-registered mobile number or email address to validate the transaction. In real applications, OTP can be repeating due to the limited length of numbers. Furthermore, an one-time pad, is one in which a key can only be used once (i.e., nonrepeating) for encrypted operations [9]. One-time pad is an encryption method in classical cryptography and has been reported by Shannon [10] to be theoretically unbreakable. Recent implementations of OTP, such as those of [11,12,13], served as references for the implementation of our own proposed key update mechanism in which we aimed to automatically execute both OTP (for changing password) and one-time pad (for nonrepeating key) in IoT devices as a network communication pair, namely D-OTP.

### 3.2. D-OTP Secure Transmission Ecosystem

**Figure 3:**
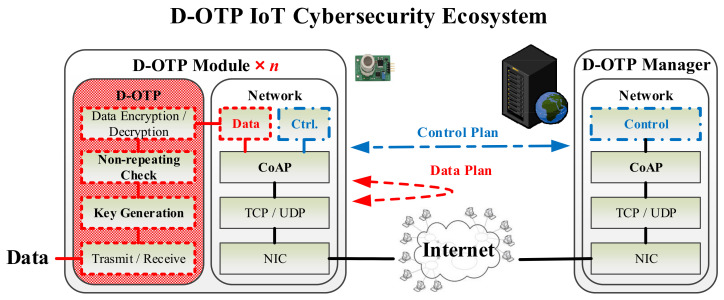
A D-OTP deployment scheme for building a secure IoT transmission ecosystem.

Figure 3 shows a deployment scheme applying D-OTP to realize a secure IoT ecosystem. The operations of the system can be divided into three planes as follows.

1. Setup Plane

**Figure 4:**
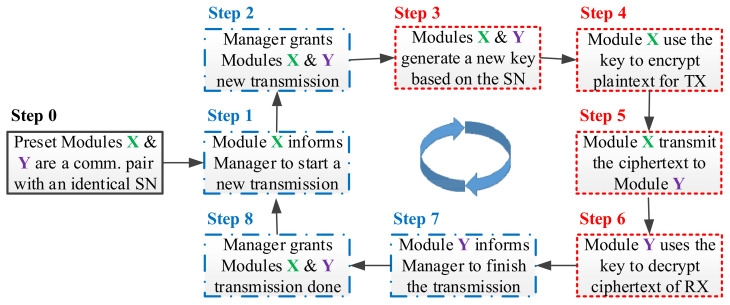
A D-OTP operation flow for activating the IoT secure transmission ecosystem.

The proposed D-OTP IoT cybersecurity system basically comprises a D-OTP manager and several D-OTP devices, and a D-OTP device shall contain at least one D-OTP module. When deploying the devices, the user can preset two modules with an identical SN as a communication pair, and the used SN is essentially nonrepeating in other communication pairs (c.f., Step 0 in Figure 4). In operations, the SN is applied to generate a nonrepeating key for each transmission (c.f., Step 3 in Figure 4). Compared with traditional PSK methods, the SN is used as an initial PSK but not transmitted via public network. In practice, the SN is recorded in a ROM, thus it can only be obtained by directly reading the ROM in the module of device. In addition, even if the SN of a certain D-OTP module is known by a third party, the SN cannot be used in hacking other device modules due to the one-time use principle.

2. Control Plane

Control plane is for managements in the system operation stage. Before a data transmission start between two paired modules, the modules must request the D-OTP manager’s grants first (c.f., Step 1 to 2 in Figure 4). When the transmission is completed, the D-OTP manager shall be informed again (c.f., Step 7 to 8 in Figure 4). The D-OTP manager can record and monitor the times of grant transmissions between the communication pair to detect any abnormal behavior such as brute-force (i.e., replay) attacks from cracking the IoT ecosystem. The control plane can be optional in deploying a D-OTP IoT cybersecurity system.

3. Data Plane

Data plane is for transfers in the system operation stage. After receiving (RX) the grant to start a data transmission, the paired modules must individually generate a new and nonrepeating key based on its SN (c.f., Step 3 in Figure 4). In the process of data transmission, the paired modules use the key to perform encryption and decryption operations, respectively (c.f., Step 4 to 6 in Figure 4). It is well known that there will be massive IoT devices in the coming future and the designed data plane of D-OTP can enable devices to directly transmit (TX) data between each other. By doing so, the D-OTP IoT cybersecurity system can distribute computing and storage resources to the endpoint devices, and switch datagrams at the network edge to reduce data transmission delays, which can be a novel realization of fog computing [35]. The data plane is mandatory for the D-OTP IoT cybersecurity system.

### 3.3. Applications of D-OTP

**Figure 5:**
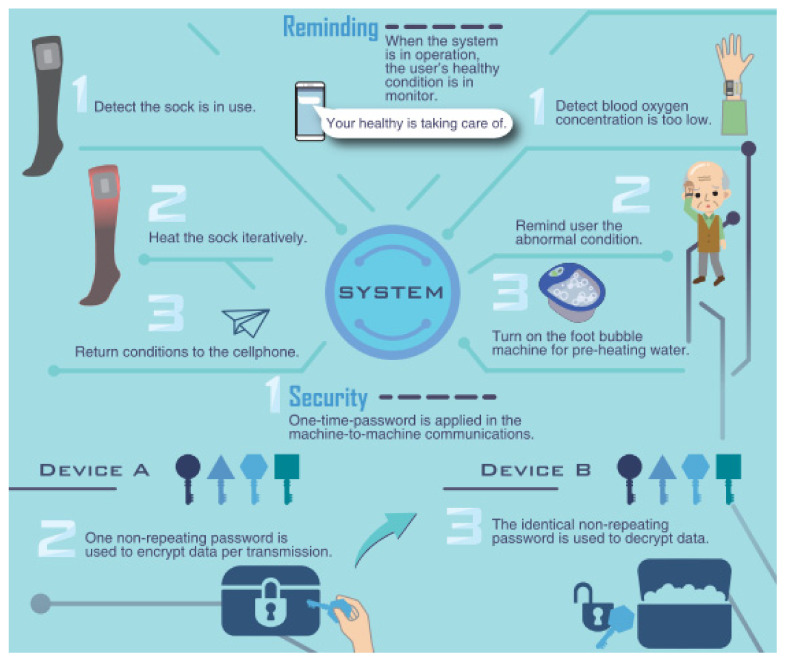
Application scenarios for healthcare IoT devices using D-OTP [36] (Supplementary Materials).

In the proposed IoT ecosystem, a mechanism has been added that repeatedly tests until a nonrepeted random number, which can be used as the key for D-OTP appications, is obtained. In practice, D-OTP is a secure transmission system suitable for IoT devices. We have applied D-OTP to several health-care devices, such as sensor bracelets, heated socks, and foot baths, as illustrated in Figure 5.

**Figure 6:**
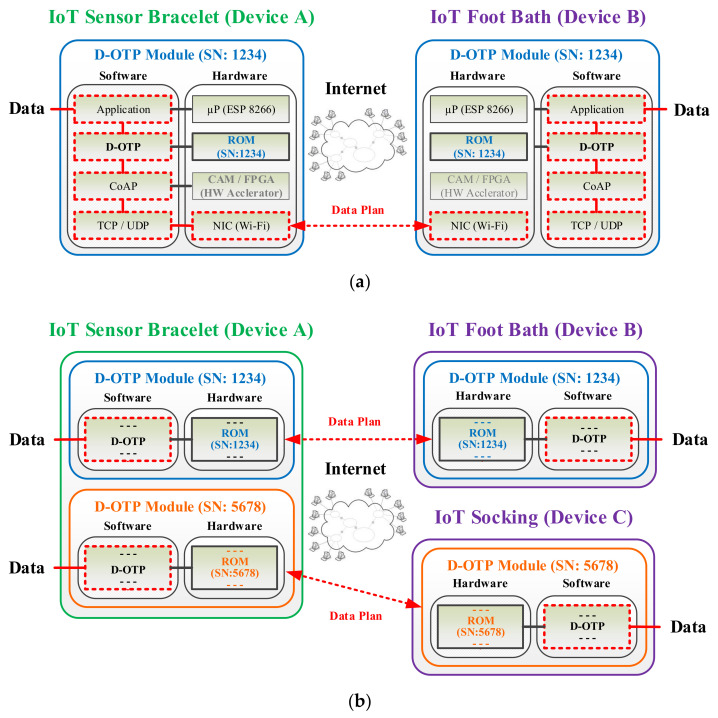
D-OTP architectures for directly transmitting data between (**a**) two or (**b**) three healthcare IoT devices.

Figure 6a illustrates a D-OTP data transmission example between two healthcare IoT devices (device A and device B). Notably, as a communication pair, the identical SN of “1234” is recorded in the ROMs of the paired D-OTP modules equipped in devices A and B, respectively. As Figure 6b shows, device A can furthermore communicate to another additional device (device C). Accordingly, device A shall be equipped with two D-OTP modules, one for the original device B (with SN “1234”) and another for added device C (with SN “5678”). In accordance with the OTP rule, the SN of device C is nonrepeating to that of device B. Afterward, the two D-OTP modules in device A can be combined into a single one with two SNs to reduce manufacturing costs.

**Figure 7:**
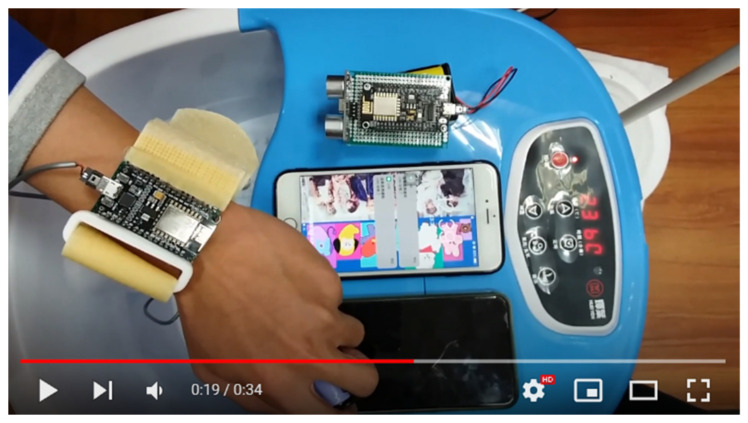
A demonstration for healthcare IoT devices using D-OTP [36].

As Figure 7 shows, we provided a Youtube video demonstration in [36] for the implemented healthcare IoT devices using D-OTP. Through the use of this system, users can benefit from the use of smart IoT devices without having their personal physiological information intercepted by a third party.

Because the life of an IoT device is limited, the total length of data transfer is finite. Therefore, in a practical design, the key blocks used to encrypt the data blocks would be nonrepeating to ensure the size (i.e., the number of bits) of a key block can reach a reasonable value. For example, a 32-bit key block can be used by D-OTP to generate 2^32^ nonrepeating key blocks, which is more than sufficient for the application target of IoT devices for healthcare purposes. If the mechanism detects that all random number combinations have been used (under use conditions that are not reasonable), our design suspends the operation of the D-OTP to prevent attempted brute-force (i.e., replay) attacks from cracking the IoT ecosystem.

### 3.4. Implementation of CoAP

We used a CoAP server C program example from libcoap [5] called CoAP server, which can be used to communicate with IoT client devices using the CoAP, with a Internet Protocol (IP) address or Uniform Resource Identifier (URI) given as an argument on the operation system shell command line. The URI determines which protocol is employed: CoAP+TCP, CoAPs, or CoAPs+TCP. Through this process, CoAPs and CoAPs+TCP are only supported when the CoAP server is developed using a DTLS [5], with the DTLS being based on TLS over the UDP socket to transfer datagrams. In our experiments, we applied the PSK mode of the CoAP server by employing the DTLS library of OpenSSL [6].

### 3.5. Implementation of PRNG

PRNG is a deterministic random bit generator, meaning the number generated by the PRNG is not truly random; it can be determined by using an initial value as a seed. For example, Barker et al. [37] presented a software-implemented algorithm for generating approximations of the properties of sequences of random numbers. Besides, Adeel et al. [3] proposed a chaotic PRNG to enable a lightweight and secure IoT transmission. By contrast, a hardware-implemented pseudorandom generator [38] was proposed that achieved a more secure cryptographically secure pseudorandom number generator. Furthermore, for an OTP to be applied and effective, a NR-PRNG such as that of [32] must be implemented. Consequently, the overhead for the nonrepeating process should be considered carefully.

### 3.6. System Execution Flow

**Figure 8:**
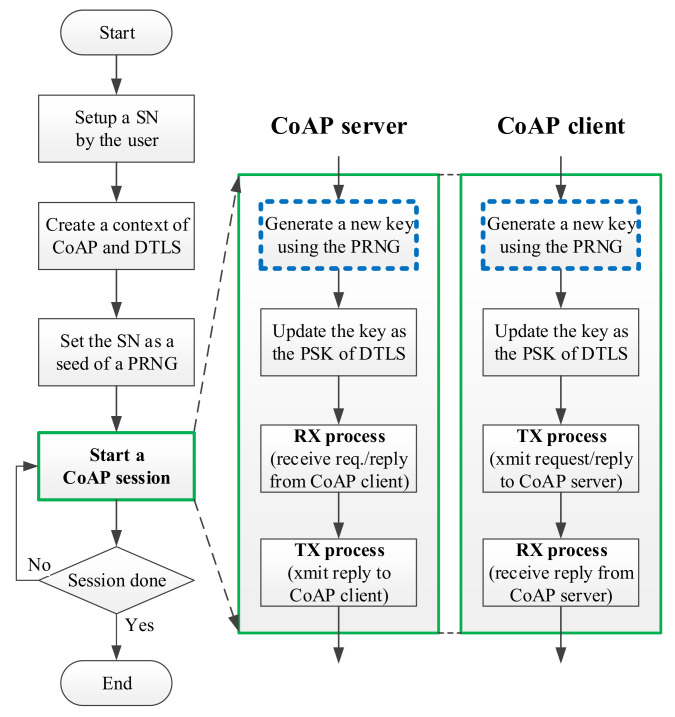
Flowchart of key updating in CoAP session.

In the original design of the CoAP server, as well as in that of the CoAP client, the PSK is assigned with a command with a “-k key” argument, where the key is the specified PSK. Accordingly, the PSK is applied for all stages of data transfers between the CoAP server and the CoAP client. As displayed in Figure 8, we use the key value assigned with the “-k key” argument in the command instead of a serial number (SN). Notably, the same SN is assigned to the session pair of the CoAP server and CoAP client. In the “start of the CoAP session” stage, a new key is created or updated by the applied PRNG, and the SN is used as a seed. For the operation of the CoAP session, the CoAP server is initiated first and waits (the “RX process” stage) for a CoAP connection request to be issued from the CoAP client (the “TX process” stage) as presented in Figure 8. For the CoAP session in our design, the key update processes are similar in the CoAP server and CoAP client; both use an identical SN as the seed of the applied PRNG or NR-PRNG to generate identical random numbers for encryption and decryption.

### 3.7. Key Update Flow

**Figure 9:**
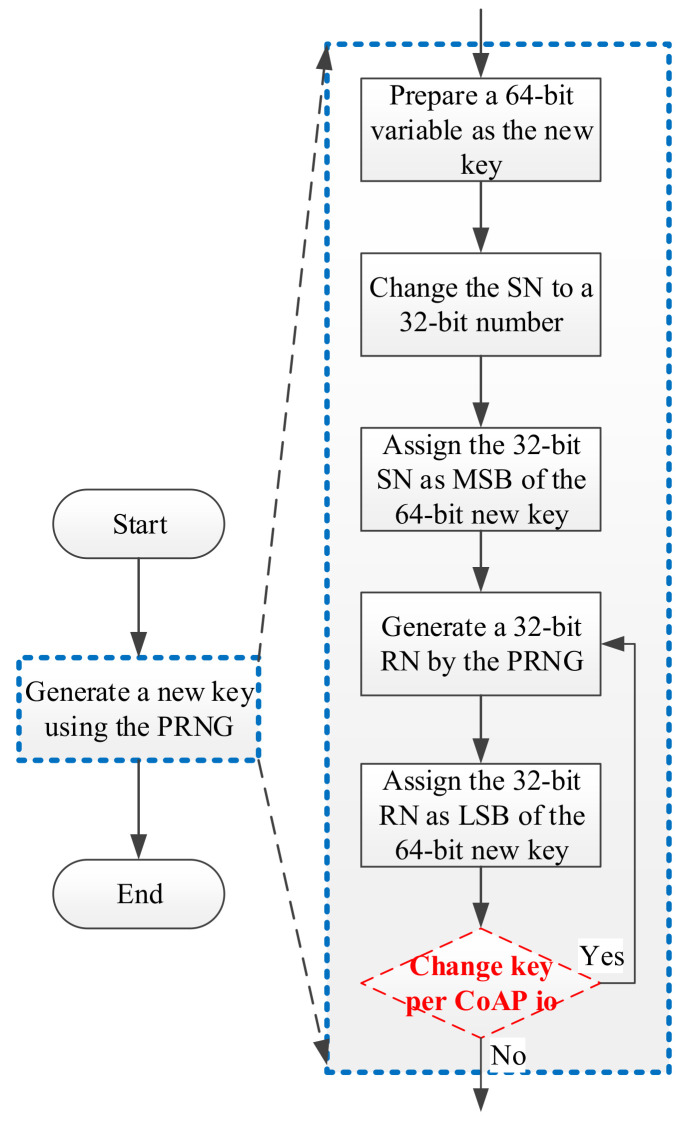
Execution flowchart of generating a new key in CoAP io.

In this section, we explain the key update flow that follows the execution flow for generating a new key (Figure 9). A similar execution flow was provided in our previous work [8].

First, the user sets an SN for the CoAP server and CoAP client. In libcoap, SNs are stored as characters according to the American Standard Code for Information Interchange (ASCII).

SN = “1234” (32-bit)(1)

The SN is subsequently changed to a 32-bit number.

SN = 1234 (32-bit)(2)

A random number (RN) is then generated by a PRNG, with the SN being used as a seed for the PRNG.

RN = 1930134286 (32-bit)(3)

The 32-bit SN and the 32-bit RN are then concatenated as a new 64-bit key, as follows:

new key = SN << 32 + RN (64-bit)(4)

new key = 5299989643264 + 1930134286 = 5301919777550 (64-bit)(5)

Finally, the key can optionly be changed per CoAP io process. A CoAP io can be regarded as one data transfer (using a UDP socket) between a CoAP server and a CoAP client. By contrast, the CoAP session is defined as a basic transmission unit of the CoAP protocol [4], one CoAP session comprises five CoAP io processes to finish the required handshakings and data transfers for the CoAP server and CoAP client communication pair. In our design, the key can be updated per CoAP session (i.e., one key is used five times for the five CoAP io processes in a CoAP session) or for each CoAP io, which guarantees each key is different in a continuing data transfer.

### 3.8. Key Synchronization Issue

**Figure 10:**
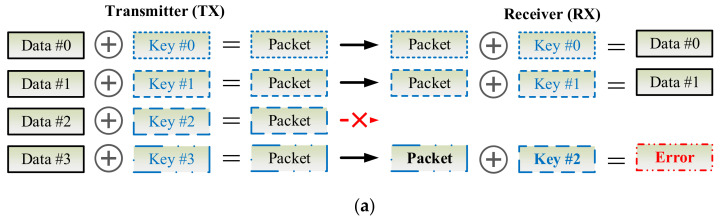

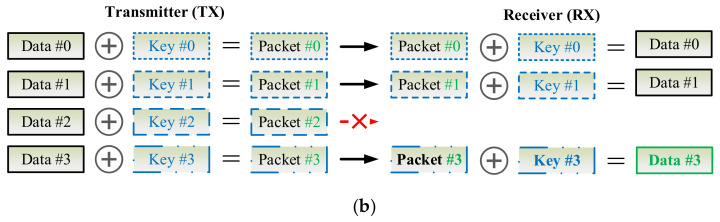
Scenarios where packets are transmitted through an unreliable transport (**a**) cause a key synchronization problem or (**b**) are patched by a basic solution to skip the key of the lost packet.

As Figure 9 shows, the key can be updated per CoAP session or for each CoAP io in the exchanges of CoAP messages between the CoAP server and client. Due to network congestion, traffic load balancing, or unpredictable network behavior, IP packets may be lost, duplicated, or delivered out of order. In an unreliable network, the proposed D-OTP mechanism possibly encounters a problem that the keys might be asynchronous between the two communication pairs as illustrated in Figure 10a, in which, the packet encrypted by key #2 is lost in transmission. In case of unawareness of the packet loss, the receiver applys key #2 to decrypt the third received packet thus leading to an error decoding result. Therefore, in order to develop D-OTP using an unreliable transport such as UDP, a field of packet serial number could be added in payload of the transmitted packets as a basic solution depicted in Figure 10b. Consequently, each received packet can be decrypted by its corresponding key.

**Figure 11:**
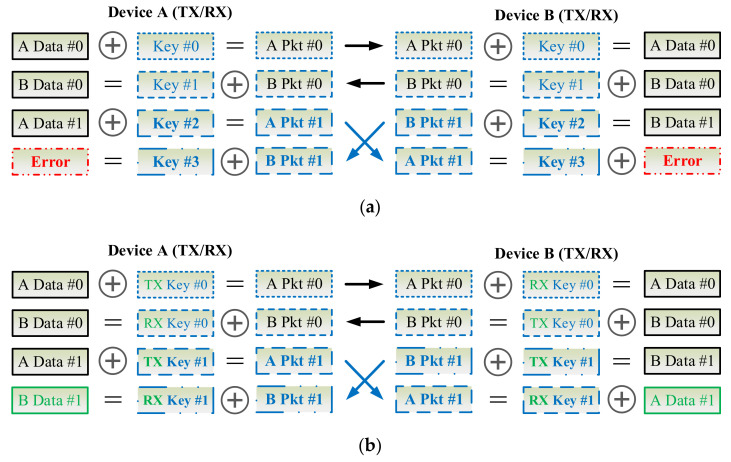
Scenarios where packets transmitted simultaneously from both devices (**a**) cause a key synchronization problem or (**b**) are patched by a basic solution to separate the keys used for TX and RX.

Another key synchronization issue is how to deal with transmissions in different directions between the communication pair. As Figure 11a shows, at the first two transmissions, the keys are changed sequentially. However, at the third transmission, device A and device B transmit (TX) packets to each other simultaneously, such a race condition leads to the error decoding results because both devices use key #3 to decrypt the received packet encryped by key #2. As Figure 11b illustrates, to deal with this problem, a simple method is that, a device shall use keys for TX and RX, separately. In other words, TX key #0 of device A is different from TX key #0 of device B, but TX key #0 of device A is identical to RX key #0 of device B, and vice versa. Therefore, a device needs two SNs to generate independent TX and RX keys. In practice, a proposal is that, to use a SN for TX and the ones’ complement of the SN is for RX, where the ones’ complement of a binary number is the value obtained by inverting all the bits in the binary representation of the number.

In an unreliable network, a general method is to employ the TCP transport, which can detect these problems, then requests re-transmission of lost data, or rearranges out-of-order data. Once the TCP receiver has reassembled the originally transmitted sequence of data, it then passes it to the receiving application. That is, TCP abstracts the communication of the application from the details of the underlying network. Although CoAP is bound to the unreliable transport of UDP, however, CoAP can still provide reliability by marking a message to be transmitted as confirmable (CON). A CON message can be retransmitted until the recipient sends an acknowledgement (ACK) message. Besides, in our experiments, CoAP messages are secured using DTLS over UDP. In practice, DTLS is TLS with added features to deal with the unreliable nature of the UDP transport [4].

## 4. Experimental Results

To verify the function and evaluate the performance of our proposed method, we performed experiments in a workstation composed of one Intel Xeon E5-1620 processor with 32 GB memory and an Ubuntu (version 18.04.3) operating system. Experimental items and performance analyses are introduced and discussed in the following subsections.

### 4.1. Executions of Libcoap

**Figure 12:**
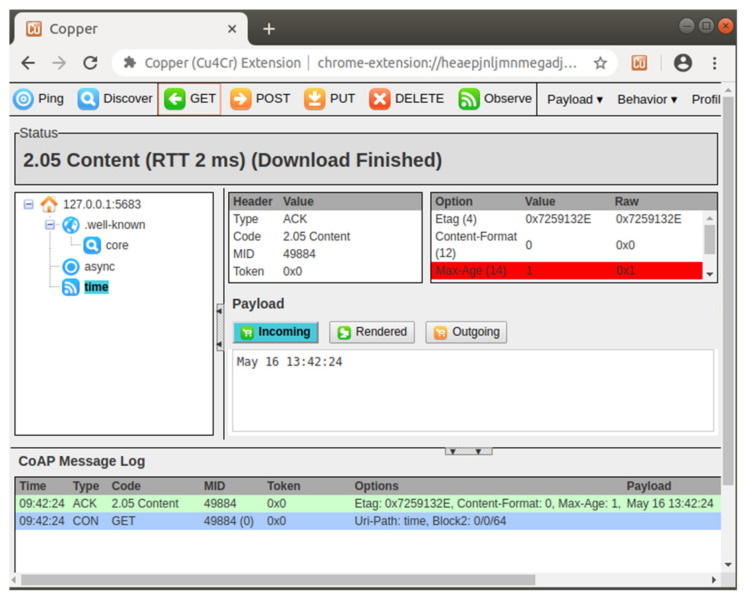
Initiation of Copper without DTLS enabled.

The implementation using libcoap includes two programs, one is CoAP server and the other is CoAP client. First, we performed the initial functions, the implemented CoAP server was enabled to await endpoint connections from multiple clients. Next, we initiated the CoAP client to receive information from the CoAP server. A Copper CoAP user-agent [39] was also applied as a graph user interface and obtained the time and date information from the identical CoAP server, as presented in Figure 12, to further assess the accuracy of the communication handshakes between the CoAP sever and CoAP client.

Furthermore, to investigate whether the security IoT ecosystem was functionally supported by OpenSSL [6], the CoAP server was enabled using an example key value of “1234” assigned with a “-k key” argument command. The CoAP client successfully received the information with the same key value of “1234” assigned with the “-k key” and “coaps” followed by an IP address as command arguments.

### 4.2. Automatic Key Update Mechanism

In operation, libcoap uses a PSK by setting an identical key for the CoAP server and multiple CoAP clients; the key is used to encrypt datagrams for all transfers that involve the CoAP server. This can lead to two security concerns: first, the identical key could be learned and misused by a malicious third party. Second, the multiple ciphertext datagrams encrypted by the identical key can enable hackers to uncover cipher rules.

To overcome these concerns, the “change key per CoAP io” phase (Figure 9) of our designed mechanism can be optionally applied to update the value of the key through the specifications “per CoAP session” or “per CoAP io” in a single CoAP session. The new key, the 64-bit number of “5301919777550” presented in Equation (5), which is dynamically updated, provides improved security compared with the original static key. The experimental results are presented in the following sections.

### 4.3. Performance Analyses for PRNG

We followed the CoAP outlined in [4] to create a CoAP session in which we used a CoAP client to obtain time and date informaton from a CoAP server; five CoAP io processes were required for the data transfers (using a UDP socket) to occur (i.e., one CoAP session contains five CoAP io processes). Accordingly, in our experiments, we implemented three key update policies and compared the performance overheads of the transfer latency performance that resulted from the different computing efforts of the policies. The first policy was to update the key “per CoAP session” (Per-Session); the key is updated using the PRNG once for each CoAP session. Therefore, one key is used five times for the five data transfers (i.e., CoAP io processes) that occurr in a single session. The second policy was to update the key “per CoAP io” (Per-IO) process. As with the per-session policy, a key for the first CoAP io is generated using the PRNG. The key is then changed by simply increasing the numbers by one for the next CoAP io (Per-IO-Inc1). However, to randomize the key used for each CoAP io beyond a simple increase of 1, we created the third policy. Through this policy, the key is updated using the PRNG once in the “per CoAP io” process (Per-IO-Prng).

**Figure 13:**
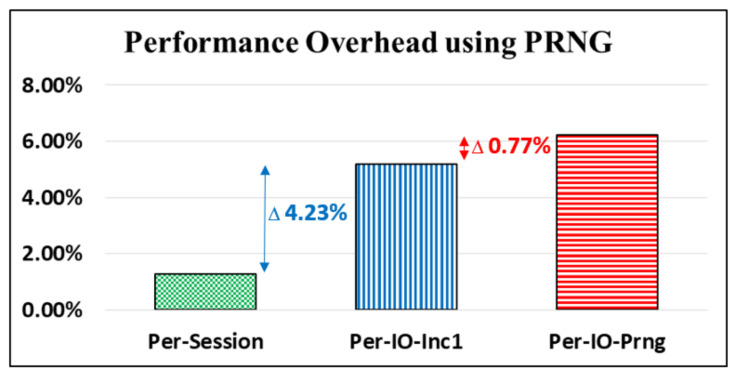
Latency performance comparisons among different key update policies.

The latency performance overheads (i.e., additional transmission latency compared with the primitive design of libcoap [5]) for the three key update policies required for the CoAP server are displayed in Figure 13. The experimental number for each policy is an average time value of the latency statistics for data transfers in 100 CoAP sessions (i.e., 500 CoAP io processes) between the CoAP server and the CoAP client. The latency overhead for Per-Session is relatively small at 1.27%. The overhead of Per-IO-Inc1 increases to 5.50% because the key must change for each data transfer. Moreover, the overhead of Per-IO-Prng is at 6.27%; notably, only 0.77% (6.27–5.50%) is added with the application of the PRNG function to more randomly change the keys and improve security. A similar experiment was provided in our previous work [8].

### 4.4. Performance Analyses for NR-PRNG

**Figure 14:**
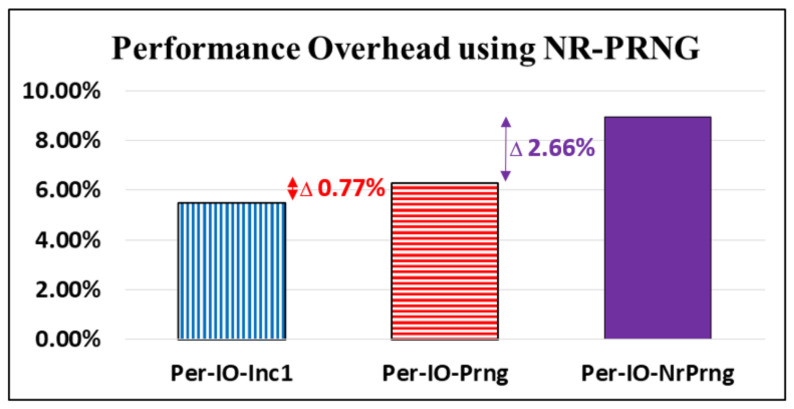
Latency performance comparisons among key update policies.

To achieve the theoretical unbreakability reported by Shannon [10], our proposed D-OTP applications required a NR-PRNG, as applied in [32]. To create performance overhead for the key without repeats, we developed a fourth policy, in which the key is updated by our designed NR-PRNG once “per CoAP io” process (Per-IO-NrPrng). The latency performance overheads for Per-IO-Inc1, Per-IO-Prng, and Per-IO-NrPrng are presented in Figure 14. The latency overhead for Per-IO-Prng is relatively small, at 0.77% (6.27–5.50%), compared with that of Per-IO-Inc1. However, the overhead of Per-IO-NrPrng increases to 8.93%, with 2.66% (8.93–6.27%) added with the implementation of the applied NR-PRNG function to change keys without repeating.

### 4.5. Performance Analyses for NR-PRNG in Mass Data Transfers

**Figure 15:**
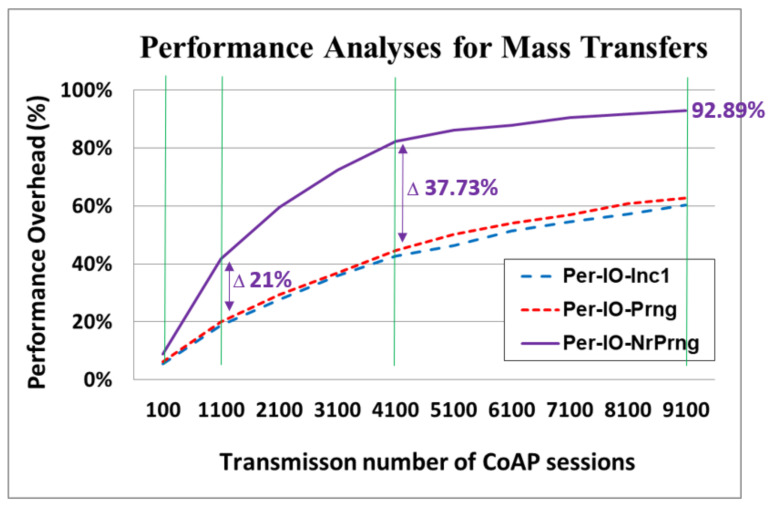
Performance analyses for mass data transfers among different key update policies.

For the Per-IO-NrPrng, a mechanism was used to repeatedly check until a nonrepetitive RN was obtained. Consequently, the overhead for the nonrepeating process warrants careful consideration. The performance analyses for mass data transfers among the different key update policies are displayed in Figure 15. The tested number of transmission ranged from 100 to 9100 CoAP sessions. The performance overhead is the percentage of additional latency caused by the applied security mechanism (the different key update policies) in the overall time period of the data transfers between the CoAP server and the CoAP client.

At 100 CoAP sessions, the performance overhead was identical to that displayed in Figure 14. For a low number of sessions, the performance overhead of Per-IO-NrPrng was higher than that of Per-IO-Prng by an acceptable 2.66% (8.93–6.27%). Notably, at 1100 CoAP sessions, the performance overhead of Per-IO-NrPrng greatly increased to 41.80%, which was considerably higher than that of Per-IO-Prng (21.83% (41.80–19.97%)). At 4100 CoAP sessions, the performance overhead of Per-IO-NrPrng further increased to 82.22%, which was 37.73% (82.22–44.50%) higher than that of Per-IO-Prng. At 9100 CoAP sessions, the performance overhead of Per-IO-NrPrng reached 92.89%, which indicates that network performance deteriorated substantially because of the NR-PRNG process. This is a critical concern that must be addressed.

### 4.6. Performance Analyses for NR-PRNG with a Long Key

In cryptography, the key length refers to the number of bits in a key used by a cryptographic algorithm; the degree of security in the cryptographic algorithm generally correlates to its key length. Therefore, the key length should be long enough to ensure that brute-force attacks that use all possible bit combinations as replay attacks would be impossible. However, an implemented system with a longer key requires more time to confirm whether the newly generated key is repetitive. As discussed in Section 3.6 of the mansucript, the key applied in our proposed IoT ecosystem is 64 bit. To investigate the performance overhead for different key lengths, we increased the key to 96 bit to accommodate the needs of our nonrepeating key generation process.

**Figure 16:**
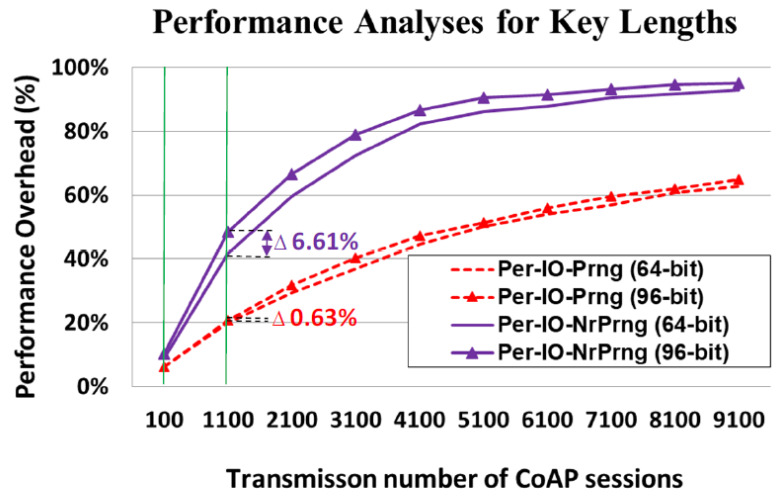
Performance analyses for 96-bit key compared with 64-bit key.

The performance analyses for different key lengths among different key update policies are presented in Figure 16. The tested number of transmissions, as with the 64-bit key, ranged from 100 to 9100 CoAP sessions. For 100 CoAP sessions, the performance overheads were trivial for both the Per-IO-Prng and Per-IO-NrPrng protocols, at 0.22% and 1.32%, respectively. For 1100 CoAP sessions, the difference between the performance overhead of the Per-IO-Prng (96-bit) and Per-IO-Prng (64-bit) was only 0.63% (20.60–19.97%); however, the gap in the performance overhead between the Per-IO-NrPrng (96-bit) and Per-IO-NrPrng (64-bit) was greater (6.61% (48.41–41.80%)). This was true for 2100 CoAP sessions and beyond. This finding indicates that application of a longer key leads to a considerable performance overhead caused by the process of the NR-PRNG.

## 5. Discussion

In comparison with the conventional IoT secure methods based on the DH key exchange algorithm, the proposed D-OTP mechanism can be applied under the present condition as shown in Figure 6. That is, the communication pair of device must be equipped with the paired D-OTP modules, in which the ROMs contain the identical SN. In practice, the paired D-OTP modules can be manufactured in the factory or preset by an engineer during deployment. In general, D-OTP is suitable for a specific application in a fixed communication system, that is the pair of D-OTP modules will only communicate with each other during their lifetime. The is the major application limitation of D-OTP compared with the commonly used DH algorithm.

However, an IoT device has a limited service life with a finite number of data transfers. In this study, we demonstrated that, when the total number of data transfers is less than 100 CoAP sessions, the poorest latency overhead incurred through application of our proposed D-OTP mechanism, which provides theoretically unbreakable security, is 8.93%. This situation is possible in several IoT applications, such as in the controls of sensor bracelets, heated socks, and foot baths (Figure 5). However, for mass data transfers, most IoT devices have limited hardware resources (e.g., microprocessors and memory). Thus, generating nonrepeating, usable D-OTP key streams in real time is challenging. The generation of the D-OTP key involves randomly selecting a nonrepeating element from a set of *n* elements. Our experimental results demonstrated that, as the number of transmissions increases, the latency performance deteriorates more rapidly. The reason for this is that, when more keys are used for transmissions, the newly generated key must be checked against all previously generated keys, which causes the time required to create a valid (i.e., nonrepetitive) key to increase exponentially. This is especially true when pure software methods, such as those of [33], are applied in experiments. Accordingly, for applications using long keys for mass data transfers, an alternative approach for hardware acceleration, such as that of [40], can offer a possible solution. The main innovations and contributions of this study are outlined as follows:

1. We propose a D-OTP mechanism that combines both OTP and one-time pad to achieve theoretically unbreakable security.
2. To the best of our knowledge, D-OTP is the first implementation of the theoretically unbreakable security in a real IoT ecosystem.
3. The proposed D-OTP mechanism can be implemented using the libcoap library, which is a popular and commonly used open-source CoAP.
4. We demonstrated that the D-OTP mechanism can be a feasible solution that provides guaranteed unbreakable security when a limited number of transmissions are being made or when latency is not a concern in lightweight M2M communications.
5. The nonrepeating key generation process may have critical performance challenges in mass data transfers, which may be a topic worth investigating in the future.

## 6. Conclusions

According to the experimental results, the traditional NR-PRNG implemented using pure software calculation, as in [33], is not sufficiently efficient to meet the performance requirements of real-time applications with mass data transmissions, as with our implemented CoAP [4], which was developed using the libcoap library [5]. However, for lightweight M2M communications in which latency is not a concern or which require a limited number of transmissions, software-based implementation of D-OTP is a feasible cost-free solution compared with the conventional security mechanisms that are not tailored to such IoT applications. To overcome the problems encountered in our study, the use of additional hardware, such as content-addressable memory (CAM), to accelerate data comparison for nonrepeating key generation could be a feasible solution [41] for adapting our mechanism for real-time applications. This may merit further investigation by future studies. The proposed D-OTP method was developed using a software-based approach. In future studies, we plan to apply a hardware-implemented acceleration to the NR-PRNG and evaluate the results in real IoT devices.

## Data Availability

Not applicable.

## Supplementary Materials

The following is available online at https://youtu.be/HnX-qpJNHjw, Application scenarios for healthcare IoT devices using D-OTP [36].

## Appendix A

Table of acronyms.

**Acronym**	**Explanation**
µP	Microprocessor
AES	Advanced Encryption Standard
ASCII	Information Interchange
AV	Audiovisual
BB84	A quantum key distribution scheme by Bennett and Brassard in 1984
CAM	Content-addressable memory
CoAP	Constrained Application Protocol
C-SGN	Cellular Serving Gateway Node
DES	Data Encryption Standard
DH	Diffie–Hellman
D-OTP	Double OTP
DTLS	Datagram TLS
eNB	Basestation of 4G-LTE
FPGA	Field-programmable gate array
gNB	Basestation of 5G
IDC	International Data Corporation
III	Institute for Information Industry
IoT	Internet of Things
IP	Internet Protocol
M2M	Machine-to-machine
NAS	Non-access stratum
NB-IoT	Narrowband Internet of Things
NIC	Network interface controller
NR-PRNG	Nonrepeating PRNG
OTP	One-time password
Per-IO	Update the key per CoAP io
Per-IO-Inc1	Update the key per CoAP io by just increase of 1
Per-IO-NrPrng	Update the key per CoAP io by nonrepeating PRNG
Per-IO-Prng	Update the key per CoAP io by PRNG
Per-Session	Update the key per CoAP session
PRNG	Pseudorandom number generator
PSK	Pre-shared key
QKD	Quantum key distribution
RC4	Rivest Cipher 4
RN	Random number
ROM	Read-only Memory
RPK	Raw public key
RSA	Rivest–Shamir–Adleman
RX	Receive
SN	Serial number
SSID	Service set identifier
SSL	Secure sockets layer
TCP	Transmission Control Protocol
TLS	Transport Layer Security
TX	Transmit
Ubuntu	An open source operating system on Linux
UDP	User Datagram Protocol
UE	User equipment
URI	Uniform Resource Identifier
Uu	Wireless interface specification of 4G-LTE
WPA	Wi-Fi Protected Access
XOR	Exclusive-OR
